# In Vivo Study of Osseointegrable Bone Calcium Phosphate (CaP) Implants Coated with a Vanillin Derivative

**DOI:** 10.3390/ph19010091

**Published:** 2026-01-03

**Authors:** Serena Medaglia, Patricia Bernabé-Quispe, Julia Tomás-Chenoll, María Cebriá-Mendoza, María Ángeles Tormo-Mas, Víctor Javier Primo-Capella, Andrea Bernardos, María Dolores Marcos, José Luis Peris-Serra, Elena Aznar, Ramón Martínez-Máñez

**Affiliations:** 1 Instituto Interuniversitario de Investigación de Reconocimiento Molecular y Desarrollo Tecnológico (IDM), Universitat Politècnica de Valencia, Universitat de València, 46022 Valencia, Spain; sermed@idm.upv.es (S.M.); maria.cebria.m@gmail.com (M.C.-M.); anberba@upvnet.upv.es (A.B.); mmarcos@qim.upv.es (M.D.M.); 2 CIBER de Bioingeniería, Biomateriales y Nanomedicina (CIBER-BBN), Instituto de Salud Carlos III, 46022 Valencia, Spain; 3 Grupo de Investigación Infección Grave, Instituto de Investigación Sanitaria La Fe (IISLAFE), Hospital Universitari i Politècnic La Fe, Av Fernando Abril Martorell 106, 46026 Valencia, Spain; patricia_bernabe@iislafe.es (P.B.-Q.); tormo_man@iislafe.es (M.Á.T.-M.); 4 Instituto de Biomecánica de Valencia, Universitat Politècnica de València, Edificio 9C, Camino de Vera s/n, 46022 Valencia, Spain; julia.tomas@ibv.org (J.T.-C.); victor.primo@ibv.org (V.J.P.-C.); joseluis.peris@ibv.org (J.L.P.-S.); 5 Unidad Mixta UPV-CIPF de Investigación en Mecanismos de Enfermedades y Nanomedicina, Centro de Investigación Príncipe Felipe, Universitat Politècnica de València, Eduardo Primo Yúfera 3, 46012 Valencia, Spain; 6 Unidad Mixta de Investigación en Nanomedicina y Sensores, Instituto de Investigación Sanitaria La Fe (IISLAFE), Universitat Politècnica de València, Av Fernando Abril Martorell 106, 46026 Valencia, Spain

**Keywords:** calcium phosphate, essential oil components, implants, antibacterial, orthopaedic injuries

## Abstract

**Background/Objectives**: Orthopaedic infections associated with implant surgery remain a major public health concern, often caused by bacterial colonization of implant surfaces. *Staphylococcus epidermidis* is among the most common pathogens involved. Developing antimicrobial bone implants that prevent infection without compromising bone regeneration is therefore essential. This study investigates the antimicrobial and osteointegrative performance of calcium phosphate (CaP) materials functionalized with vanillin, an essential oil component with known antimicrobial properties. **Methods**: Commercial CaP regenerative materials were covalently coated with vanillin. Antibacterial activity was evaluated against *Staphylococcus epidermidis* RP62A using viability assays. In vivo osseointegration was assessed in New Zealand female rabbits implanted with vanillin-coated and uncoated CaP scaffolds. **Results**: Vanillin-functionalized CaP scaffolds exhibited strong bactericidal activity at 24 h and bacteriostatic effects at 48 h at a concentration of 10 mg/mL. In vivo analyses showed no significant differences in osseointegration between vanillin-coated implants and control CaP materials. **Conclusions**: Vanillin-functionalized CaP materials maintain a high safety profile without impairing bone integration, supporting their potential use in clinical applications.

## 1. Introduction

Orthopaedic injuries and associated pathologies represent a major public health problem worldwide, as well as a serious global burden of disability and suffering. These orthopaedic conditions or injuries often require surgery and permanent, temporary or biodegradable medical devices, including various natural or synthetic biomaterials that can replace or repair various tissues. The choice of material for the medical device to be implanted plays a crucial role in the success of the orthopaedic procedure. The development of calcium phosphate ceramics and other related biomaterials for bone grafting has resulted in better control of the biomaterial resorption and bone replacement process. In particular, bioceramics have been extensively studied, and many ceramic-based devices have been marketed for their suitability as bone graft substitutes or prosthesis liners [1]. Developed bioceramics mainly include glass-ceramics (bioactive glasses) and calcium phosphates (such as calcium hydroxyapatite (HA), tricalcium phosphate, and biphasic calcium phosphate) [2]. Specifically, calcium phosphates (CaPs) are extensively used in orthopedics and dentistry, particularly in the fabrication of prosthetic surface coatings and bone cements for the treatment of bone defects. Their suitability as bone graft substitutes primarily arises from their chemical and structural similarity to the mineral phase of natural bone, which is composed of approximately 60% calcium phosphate [3,4]. Commercially available CaP formulations exhibit excellent bioactivity, controlled biodegradability, and biocompatibility [5,6,7] and in this work, we focused on using β-tricalcium phosphate materials (β-Ca_3_(PO_4_)_2_) (vide infra). Biocompatibility is one of the mandatory requirements for the clinical use of biomaterials in orthopaedics [8]. In fact, biological rejection of an implant leads to an inflammatory response mediated by immune cells and may require implant removal [9,10]. Furthermore, CaP-based materials have proven to be highly bioactive, which means they can participate in specific biological reactions that help bone regeneration [11].

From another point of view, in orthopaedic surgery bone infections associated with the colonization of implant surfaces by pathogenic microorganisms remain a major clinical concern [12]. Indeed, septic failure represents the second leading cause of prosthetic failure, accounting for approximately 18.4% of reported cases [13]. Once adhered to the implant surface, bacteria can proliferate and form biofilms, which confer increased resistance to antibiotic treatments and significantly impair the post-operative healing process [14]. In fact, biofilm formation plays a crucial role in the pathogenesis of orthopaedic implant-associated infections [15]. The biofilm protects the bacteria from both the host immune response and antibiotics, making treatment and eradication of this condition extremely difficult, considering that the bacteria in biofilms are 100–1000 times less susceptible to antibiotics with respect to planktonic bacteria [16]. In this scenario, *Staphylococcus epidermidis* is one of the major pathogens associated with orthopaedic implant-associated infections [17]. *S. epidermidis* is a ubiquitous member of the human skin microbiome and lacks aggressive virulence properties [18]. However, *S. epidermidis* can be considered an opportunistic pathogen and many infections are derived from the same strains that inhabit the skin [19,20]. One of the key virulence factors contributing to *Staphylococcus epidermidis* infections is its ability to form biofilms on the surfaces of a wide range of materials [20]. Biofilm development generally proceeds through three main stages: initial attachment, maturation, and dispersion. During the initial stage, bacterial cells adhere to the biomaterial surface or surrounding host tissue; this adhesion subsequently stabilizes, leading to the formation of microcolonies and biofilm maturation. In the final stage, bacterial cells may detach from the established biofilm and disseminate to other regions of the implant surface, thereby promoting infection spread [16] and potentially leading to reinfection episodes [21]. In short, primary stability, promotion of rapid osseointegration and antimicrobial properties of the implant are sought in cases of permanent implants applied to the bone. Based on the above, there is a growing interest in developing new bioceramics with antimicrobial properties against the development of bone infection processes [22,23]. Although the oral administration of antibiotics can be a solution for infections, unfortunately, biofilm formation on the materials’ surface requires high doses of drugs for infection eradication [24,25]. Consequently, the overuse of antimicrobials can lead to antibiotic resistance or kidney and liver complications [26]. To this end, much research has been developed on the potential use of natural compounds. The general focus has been on studying the versatile properties of essential oil components (EOCs) as a possible alternative to synthetic antimicrobial agents [27,28]. These compounds are naturally synthesized by plants and have been reported to exhibit a broad spectrum of biological activities, including antimicrobial, antiviral, antifungal, and antioxidant properties [29,30]. Many EOCs (vanillin, cinnamaldehyde, thymol, eugenol, etc.) are widely used in the medical and food industries. However, due to their high volatility, hydrophobicity and reactivity, EOCs quickly lose activity [31], which makes their application in the therapeutic field difficult [32]. For this reason, drug delivery devices and antibacterial surfaces have been extensively developed using EOCs to achieve local action [33,34]. Several studies have been conducted to attach essential oils to specific surfaces to enhance and/or prolong their antimicrobial effect [35,36].

Considering the aforementioned studies, in this work, we coat a commercial bone regenerative material with an essential oil derivative to combine the bone regeneration capability of the material with the antimicrobial action of EOC. Commercially available CaP granules (Surgibone©) are used for this purpose, and vanillin is chosen as antimicrobial EOCs. Vanillin was selected for its combination of high biocompatibility, lower cytotoxicity compared with other essential oils (e.g., eugenol, carvacrol, thymol) https://doi.org/10.1016/j.heliyon.2023.e19280; https://doi.org/10.1080/15376516.2021.1940408; https://doi.org/10.1016/j.fct.2020.111858, and chemical suitability for covalent surface immobilization. Its intrinsic aldehyde group enables direct attachment to amine-functionalized surfaces via Schiff base formation, providing a simple and reproducible immobilization strategy. Unlike other essential oils, which may require chemical modification (https://doi.org/10.1016/j.foodchem.2017.04.118), or lose activity upon anchoring (https://doi.org/10.1016/j.ijfoodmicro.2011.07.034), vanillin retains antimicrobial function after immobilization because its phenolic hydroxyl group remains exposed. Furthermore, vanillin’s regulatory status under REACH supports its safe use and potential for clinical or medical applications.

To immobilize the vanillin on the surface of the solids, the natural product is reacted with 1,5-pentane diamine to obtain a derivative which is subsequently grafted onto the outer surface of the CaP granules. The new material is evaluated against *Staphylococcus epidermidis* RP62A, a Gram-positive bacterium involved in infections of implants [37,38]. Then in vivo assays are performed on New Zealand female rabbits. The animals underwent surgery to place experimental and control materials in the median region of the distal femoral condyle. Post-operatively, biocompatibility and osseointegration ability of the experimental material are assessed.

## 2. Results and Discussion

### 2.1. Synthesis and Characterization of the Functionalized CaP Material

**Scheme 1:**
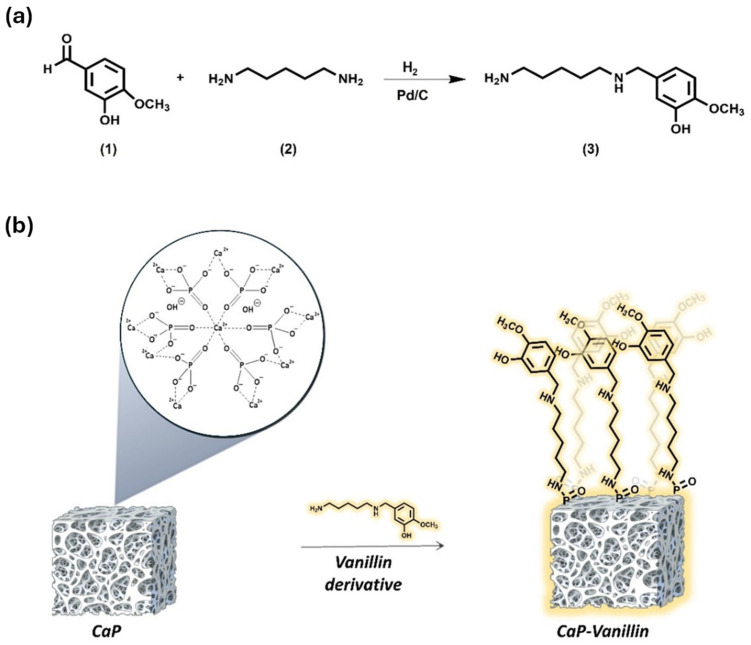
(**a**) Synthetic protocol to prepare the vanillin derivative (**3**); (**b**) Representation of CaP-Vanillin synthesis.

This study focuses on modifying commercial CaP granules with a vanillin-derived compound (**3**) (Scheme 1a), aiming to integrate the osteoconductive characteristics of calcium phosphates [39] with the antimicrobial potential of vanillin-based essential oils. Vanillin was selected as the essential oil component due to its well-established antibacterial properties. In the first step, vanillin was reacted with 1,5-pentanediamine to obtain the corresponding Schiff base (compound **2**) that was further reduced by a chemical hydrogenation reaction using H_2_ and palladium (Pd/C) as a catalyst. This allows the vanillin derivative (**3**) to be obtained and fully characterized (see Supplementary Information). In a second step, CaP was functionalized with the vanillin derivative (**3**) by phosphoramide formation using EDC as coupling agent to obtain the solid CaP-Vanillin (see Scheme 1b).

**Figure 1:**
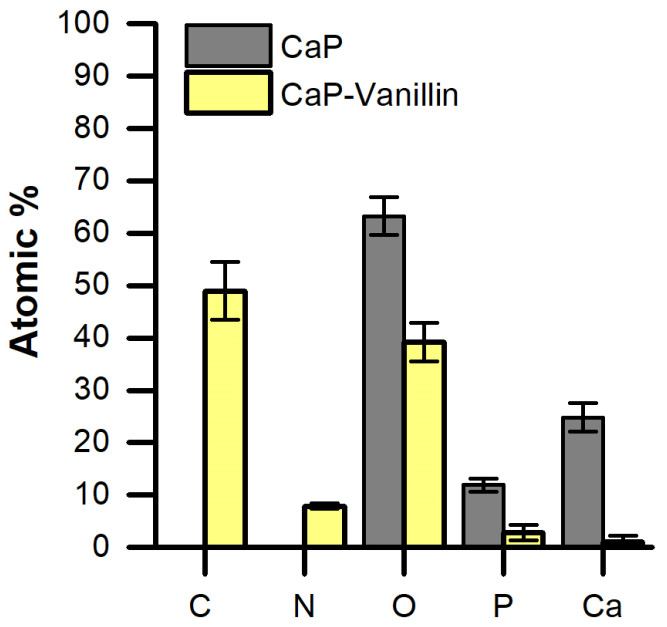
HR-FESEM-EDX analysis of CaP (grey) and CaP-Vanillin (yellow) surfaces.

**Figure 2:**
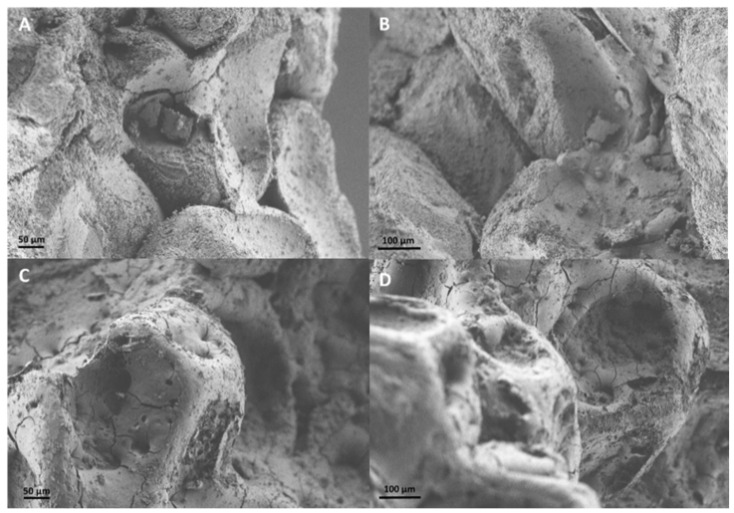
HR-FESEM images of CaP (**A**,**B**) and CaP-Vanillin (**C**,**D**) surfaces.

The prepared materials were characterized using different techniques. The TGA showed that the CaP-Vanillin material contains 2.9 mg of organic matter per gram, corresponding to a surface site density of 12.2 µmol g^−1^ The analysis obtained with HR-FESEM-EDX (Figure 1) of the CaP and CaP-Vanillin shows, in the case of functionalized materials, the presence of nitrogen, the decrease in the percentage of P (12% for CaP and 3% of CaP-Vanillin), Ca (25% for CaP and 1% of CaP-Vanillin) and O (63% for CaP and 39% of CaP-Vanillin), and increase in the percentage of C (0% for CaP and 49% of CaP-Vanillin), confirming the immobilization of the vanillin derivative **3** on the CaP. Moreover, HR-FESEM images of CaP and CaP-Vanillin (Figure 2) demonstrated that the CaP surface before and after functionalization with the vanillin derivative was similar, indicating that the functionalization process did not change the CaP structure at the micrometric level. In addition, IR was also used to follow the functionalization. Comparison of the spectra of CaP and CaP-Vanillin revealed peak shifts located at 1049, 634 and 606 cm^−1^ attributed to the formation of specific phosphoramide bonds between CaP and derivative **3** [40].

### 2.2. Antibacterial Capacity of CaP Material Functionalized with Vanillin

**Figure 3:**
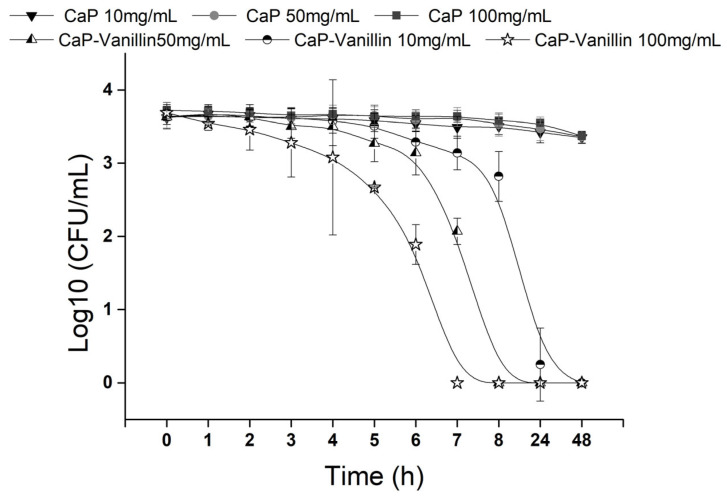
Time-kill curve for *S. epidermidis* RP62A exposed to CaP or CaP-Vanillin at 10, 50 and 100 mg/mL.

The antimicrobial activity of the prepared materials was tested against *S. epidermidis*. *S. epidermidis* strains are closely related to most orthopaedic infections, and it was used to study the antibacterial ability of CaP-Vanillin by time-kill assay. In this experiment, different concentrations of Cap-Vanillin (10, 50 and 100 mg/mL) were used to test the bactericidal activity of each concentration. In all three cases, a bactericidal effect was achieved, but the main difference between them was the time required to achieve it (Figure 3). In the case of the highest concentration (100 mg/mL), a reduction in bacterial viability was observed after seven hours of treatment. On the other hand, using 50 mg/mL and 10 mg/mL, the bactericidal effect was observed after 24 h and 48 h of treatment, respectively. That is to say, the bactericidal effect of this material is dose-dependent. To relate these results to an effective drug concentration, the organic loading determined by TGA (2.9 mg of organic matter per gram of functionalized solid) was used to calculate the equivalent concentration of the immobilized vanillin derivative. Consequently, the solid concentrations of 10, 50, and 100 mg/mL correspond to effective vanillin derivative concentrations of approximately 29, 145, and 290 µg/mL, respectively. This normalization highlights that significant bactericidal activity is achieved at relatively low effective doses of the active motif. On the other hand, equivalent amounts of CaP support (10, 50 and 100 mg/mL) were used as controls. In all three cases, there was no reduction in viability, so CaP has no antimicrobial activity. These results indicate that the antibacterial activity of the functionalized CaP-Vanillin material is due to vanillin and better results are obtained by using a higher concentration of CaP-Vanillin. The intended use of CAP-Vanillin is for localised treatment, which allows for higher concentrations of the antibiotic (i.e., vanillin) to be used. In addition, with this type of formulation, the systemic toxicity of the compound would be reduced [41], which is advantageous over orally or parenterally administered antibiotic therapy.

### 2.3. Antibiofilm Capacity of CaP Material Functionalized with Vanillin

**Figure 4:**
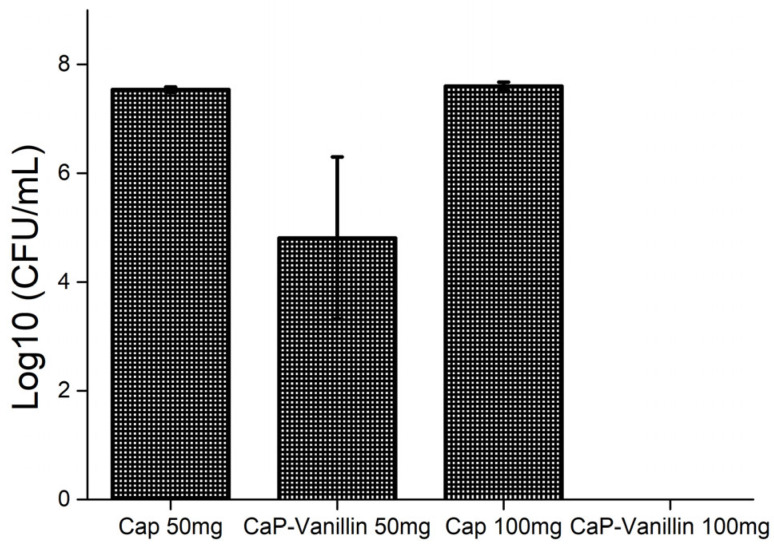
Effect of CaP-Vanillin on the biofilm formation of *S. epidermidis* RP62A in comparison with CaP at different concentrations (50 mg/mL and 100 mg/mL). Viable cells quantification of the biomass of a 48 h biofilm.

**Figure 5:**
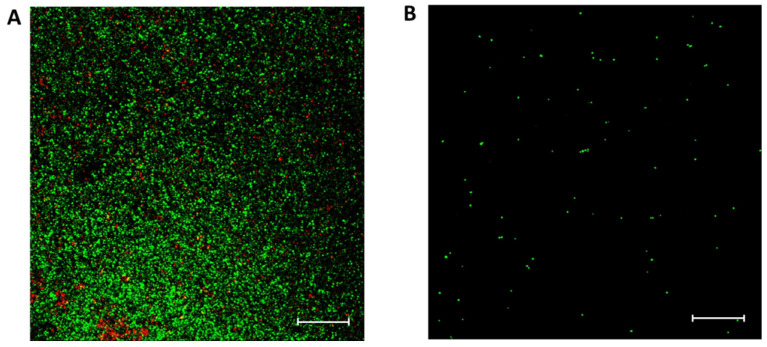
Confocal microscopy images (40× oil objective) of LIVE/DEAD^®^-stained biofilm of *S. epidermidis* RP62A (Waltham, MA, USA). The bacteria were grown in TSBG for 48 h with CaP (**A**) and CaP-Vanillin (**B**) at 100 mg/mL concentration. Images reveal that mature biofilm development is inhibited by CaP-Vanillin.

Avoiding biofilm formation is a strategic step in preventing infections related to prosthetic devices [42]. Consequently, the ability of CaP-Vanillin to prevent biofilm formation of the high biofilm-forming *S. epidermidis* strain RP62A was also studied. From a quantitative perspective, CaP-Vanillin showed high ability to reduce biofilm formation after 48 h of incubation of *S. epidermidis* RP62A. CaP-Vanillin achieved a 7-log reduction in viable *S. epidermidis* biofilm cells compared to CaP at a concentration of 100 mg/mL, producing a 2-log reduction at a concentration of 50 mg/mL (Figure 4). The average lethality of *S. epidermidis* in biofilm produced by CaP-Vanillin was 98.9% and >99.9% with a concentration of 50 mg/mL and 100 mg/mL, respectively in comparison with CaP. In addition, the effect of CaP-Vanillin on preventing biofilm formation was studied using confocal laser microscopy; in confocal images significant differences were observed between the biofilm formed in the control (CaP) and the biofilm formed in presence of CaP-Vanillin. As shown in Figure 5, *S. epidermidis* formed robust biofilms when cultured in the presence of CaP. In contrast, biofilm formation was inhibited in presence of 100 mg/mL CaP-Vanillin. These results showed that CaP-Vanillin strongly prevented the biofilm formation of *S. epidermidis*.

Vanillin is a molecule used as an additive in the food industry and is included in the list of food additives generally regarded as safe (GRAS) [43,44]. In addition, vanillin is a natural compound that has been reported to exhibit, among others, antimicrobial activity and antibiofilm activity, partly through disruption of bacterial membrane integrity, increased membrane permeability, and interference with quorum sensing mechanisms [43]. Similarly to other essential oil compounds (EOCs), vanillin has been explored as an alternative to chemically synthesized antibiotics. However, a key limitation of EOCs is their high volatility, which can be addressed by covalent immobilization onto surfaces [45]. Surface anchoring also provides higher local density, more homogeneous distribution, and greater stability and functionality of the attached molecule [46]. In the present system, vanillin is covalently immobilized onto CaP surfaces via its aldehyde group. Although covalent attachment prevents diffusion into the surrounding medium, the phenolic hydroxyl group remains exposed at the surface, preserving antimicrobial activity in a contact-mediated manner. The stability of this covalent anchoring—mediated by the robust phosphoramide bond and the chemically stable secondary amine formed during synthesis—prevents the leaching of the vanillin derivative into the surrounding medium. This chemical stability, combined with the rigorous washing protocol employed during synthesis to remove any physisorbed molecules, ensures that the observed bactericidal and antibiofilm effects are driven strictly by the surface-exposed phenolic moieties and not by eluted species. Thus, bacteria coming into direct contact with the functionalized surface are inactivated, while the molecule remains stable and non-leaching. Such a strategy combines the chemical robustness of covalent attachment with the biological activity of vanillin and represents a practical approach to confer long-lasting antimicrobial properties to CaP surfaces. In the literature, there are some works in which the surface of CaP is modified in order to add antimicrobial activity. These modifications are by binding Ag or W nanoparticles [47,48], by using lipid nanoparticles carrying the EOC carvacrol [49], or by coating the CaP surface with a layer of tea tree oil [50]. The surface modification can either inhibit bacterial adhesion and biofilm formation (antiadhesive or anti-microfouling action) or directly kill adhered bacteria (bactericidal action) [51]. In most of these examples, antimicrobial activity relies on the controlled release of the active compound, which produces only a transient local effect, and the EOCs do not remain active over time. In contrast, the covalent immobilization of vanillin in the present work ensures that both bactericidal and antibiofilm activities are maintained at the surface, as demonstrated in the results for CaP-Vanillin.

### 2.4. In Vivo Study

Orthopaedic injuries and associated pathologies are a major public health problem, so there is a need to develop new materials with anti-inflammatory and antimicrobial properties that present high biocompatibility, bioactivity and biodegradability. Nowadays, poly(methyl methacrylate) (PMMA) bone cement is the gold standard biomaterial for local antibiotic therapy in orthopaedics and has been used for over 35 years for both prophylaxis and treatment of bone infection [21,52,53]. However, this material has some limitations regarding the antibiotics that can be used. For instance, its polymerization reaches high temperatures, so the antibiotics to be administered embedded in this material must be thermolabile [54]. On the other hand, certain antibiotics are incompatible with PMMA due to their ability to scavenge free radicals and impair PMMA polymerization [21,55]. It is also important to note that PMMA is not biodegradable. Therefore, it must be eliminated from the body at some point, which would require a second surgical intervention and the associated risks [21].

In this scenario, there is a need to find new therapeutic materials that are biodegradable to avoid surgery and that can also be absorbed by the body. One of the main candidates are materials based on calcium phosphates [56,57]. CaP-based materials do not have antimicrobial activity, so adding some compound that presents this activity, such as EOCs, makes it possible to obtain multifunctional biomaterials [58]. In fact, it has been previously demonstrated that CaP particles present good biocompatibility, bioactivity, and biodegradability [5,6,7]. Moreover, a previous study [59], demonstrated that the functionalization of CaP particles with vanillin did not present any cytotoxic effect on fibroblast-like cells and that it did not modify the activity of osteoblast-like cells, which grew even on the material. The subsequent step before testing efficacy in complex sepsis models, it is a regulatory and scientific requirement to demonstrate that the new material allows for normal osseointegration. On this basis, we further tested herein if functionalization of CaP particles with vanillin affects osseointegration in vivo. For this purpose, in vivo studies were carried out in New Zealand rabbits (*n* = 6 samples per group) that were submitted to surgery to place CaP-Vanillin and CaP (as control) in the median region of the distal femoral condyle having bone defects of 4 mm in diameter and 6 mm in depth. Animals were divided into four groups. A two-factor analysis (Functionalization × Time) was implemented to evaluate the bone healing process.

**Figure 6:**
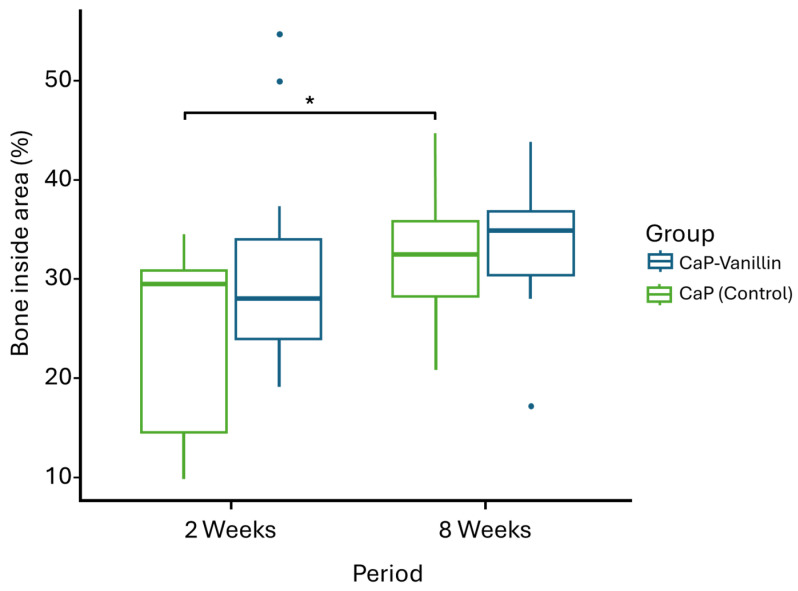
Temporal evolution of bone area inside percentage (* *p* < 0.0263) (*n* = 6).

Figure 6 shows the quantitative results of bone formation (bone area inside for CaP-Vanillin and control (CaP) samples at 2 and 8 weeks). At 2 weeks, no significant differences were found between groups (*p* = 0.101; mean difference = −7.16; 95% CI: −15.82 to 1.50), and this parity was maintained at 8 weeks (*p* = 0.618; mean difference = −1.48; 95% CI: −7.56 to 4.59). Regarding the temporal factor, a significant increase in bone area was exclusively observed for CaP-Vanillin samples (*p* = 0.0263; mean difference = +8.08), whereas the control group showed no significant growth over time (*p* = 0.535). Comprehensive data, including *p*-values, 95% CIs, and effect sizes, are detailed in Table 1. All rabbits recovered well from surgery, despite no antimicrobial treatment being used in any of the groups. In summary, with respect to temporal evolution, CaP-Vanillin samples had significant growth of bone area between 2 and 8 weeks, achieving an amount of bone growth compared to control conditions, indicating that the functionalization with vanillin does not impede bone growth. The observed temporal increase in bone area for the CaP-Vanillin group (*p* = 0.0263) can be explained by the specific biological mechanisms through which vanillin modulates osteoblast activity. Recent studies [60] have demonstrated that vanillin promotes osteoblast differentiation and maturation by upregulating the BMP2/Smad1/5/8 and RUNX2 signaling pathways, which are essential regulators of bone matrix mineralization. Furthermore, vanillin has been shown to enhance F-actin polymerization and osteoblast migration, morphological changes that accelerate the colonization of the scaffold surface during the initial stages of bone repair. Beyond its osteogenic potential, vanillin exerts a significant antioxidant effect, reducing reactive oxygen species (ROS) accumulation and protecting mitochondrial integrity. This protective capacity prevents osteoblast apoptosis under conditions of oxidative stress, thereby ensuring a higher density of viable bone-forming cells within the implant site. Consequently, the covalent functionalization of CaP with vanillin creates a pro-osteogenic environment that supports the significant bone growth observed in our in vivo model.

The potential for clinical translation of vanillin-functionalized CaP scaffolds is supported by their chemical stability, manufacturing scalability, and favourable regulatory profile. The covalent immobilization of the active motif through robust phosphoramide bonds prevents the leaching of the vanillin derivative into the surrounding medium, ensuring a stable, contact-mediated antimicrobial effect that avoids systemic toxicity. From a manufacturing perspective, the synthetic protocol for the vanillin derivative is highly efficient, achieving a 99% yield, which facilitates its industrial scale-up. Furthermore, vanillin is a natural compound with GRAS status and is already regulated under REACH, which significantly reduces regulatory hurdles for clinical approval compared to synthetic antibiotics. Unlike the current gold standard PMMA bone cement, these functionalized scaffolds are biodegradable, potentially eliminating the need for a second surgical intervention to remove the material. Finally, the material demonstrated excellent compatibility with sterilization protocols; as shown in our in vivo study, the functionalized scaffolds maintained their osseointegrative capacity over 8 weeks, with no significant differences compared to commercial controls (*p* = 0.618), confirming that the functionalization process does not compromise biological performance.

## 3. Materials and Methods

### 3.1. Reagents, Bacterial Strain and Growth Conditions

The chemicals vanillin, imidazole, and palladium on carbon (Pd/C) were provided by Sigma (Sigma-Aldrich Química S.L., Madrid, Spain). Also, 1,5-pentanediamine and N-(3-dimethylaminopropyl)-N’-ethylcarbodiimide hydrochloride (EDC) and palladium on carbon (Pd/C) were provided by Sigma (Sigma-Aldrich Química S.L., Madrid, Spain). The solvent ethanol (extra pure), dichloromethane, diethyl ether, glucose and acetone were purchased from Scharlab (Barcelona, Spain). LIVE/DEAD^TM^ BacLight^TM^ Bacterial Viability kit were provided by Thermo Fisher Scientific (Waltham, MA, USA). Propofol Lipuro was purchased from BBraun (Melsungen, Germany). Sodium pentobarbital, Dolethal, was purchased from Vetoquinol (Lure, France).

Chemicals Commercial Surgibone© material was purchased from Surgival (Paterna, Spain) in the form of granules (1–2 mm) composed of 75% hydroxyapatite and 25% β-tricalcium phosphate.

Antibacterial activity and the ability to prevent biofilm formation were evaluated against Gram-positive bacteria, *S. epidermidis* RP62A (ATCC35984) (RP62A) strain from ATCC (Manassas, VA, USA). RP62A is a methicillin-resistant strong biofilm-forming clinical strain. Stocks were stored in Tryptone Soya Broth (TSB) (Scharlab SL, Barcelona, Spain) containing 20% glycerol (Scharlab Barcelona, Spain SL) at −80 °C. Before analysis, each isolate was subcultured twice on Tryptone Soya Agar (TSA) (Scharlab SL, Barcelona, Spain) plates to ensure viability.

### 3.2. Synthesis of Vanillin Derivative *(**3**)*

The vanillin derivative was synthesised according to the previously described (Polo et al., 2018 [59]) with minor variations. Briefly, a two-step synthetic procedure was used to prepare the vanillin derivative. In the first step, 760 mg (5 mmol) of vanillin (compound **1** in Scheme 1a) was suspended in 15 mL of dichloromethane. Then 616 µL (5 mmol) of 1,5-pentane diamine in dichloromethane (25 mL) was added dropwise to the mixture under vigorous stirring (see Scheme 1a). The mixture was stirred for 1 h, diethyl ether (40 mL) was added over the suspension, and the pale yellow solid was filtered off (compound **2** in Scheme 1a). In the subsequent step, 0.7 g of the obtained solid was dissolved in 18 mL of ethanol, followed by the addition of 184 mg of Pd/C. The reaction vessel was then purged with nitrogen to remove oxygen, after which a hydrogen flow was introduced. The mixture was stirred for 3 h under inert conditions. Finally, the suspension was filtered and concentrated under reduced pressure, yielding compound **3** (Scheme 1a) as a pale-yellow solid with a 99% yield.

### 3.3. Preparation of CaP Functionalised with Vanillin Derivative (CaP-Vanillin)

For the functionalization of the granules (CaP) [59] the vanillin derivative (**3**) (523 mg) was dissolved in a solution of imidazole in water (10 mL, 0.1 M). On the other hand, the phosphate groups of CaP (300 mg) were activated by immersion of the material into a solution of EDC (1.25 g, 6.52 mmol) in Phosphate-buffered saline (PBS)-EDTA 0.01 M buffer (10 mL). Subsequently, the solution of derivative (**3**) was added to the granules, and the samples were stirred for 24 h at 37 °C. The resulting functionalized solid, CaP-Vanillin, was filtered, washed several times with water and acetone, and vacuum dried to remove any unreacted vanillin derivatives, ensuring that only the derivative covalently bound to CaP remained on the surface. Finally, sterilization of all samples prior to be used in characterization, in vitro and in vivo studies was performed by Aragogamma S.L. (La Roca del Vallés, Spain) through gamma irradiation (25 kGy).

### 3.4. Characterization Methods

The synthesized materials were characterized using high-resolution field emission scanning electron microscopy (HR-FESEM), energy-dispersive X-ray spectroscopy (EDS), thermogravimetric analysis (TGA), and infrared spectroscopy (IR).

HR-FESEM imaging and EDX measurements were performed with a Gemini SEM 500 microscope (Zeiss, Oberkochen, Germany, Oxford Instruments, Abingdon, UK), employing an SE2 detector at 1 kV, a working distance of 3.7 mm, and a standard aperture of 30 µm. TGA analyses were conducted on a TGA/SDTA 851e Mettler Toledo instrument (Greifensee, Switzerland) under an oxidizing atmosphere (air, 80 mL/min), following a heating program of 10 K/min from 393 K to 1273 K, with an isothermal step at the final temperature for 30 min. The functionalization of the solid was monitored by IR spectroscopy using a Bruker Tensor 27 FTIR system (Billerica, MA, USA). The vanillin derivative was further characterized by nuclear magnetic resonance (NMR), with ^1^H and ^13^C spectra recorded on a Bruker Advance III (400 MHz, Billerica, MA, USA) using deuterated solvents.

### 3.5. Antibacterial Activity

The inoculum was prepared by suspending 1 to 3 colonies in PBS pH 7.4 from a 24 h culture and adjusting cell turbidity to 0.5 McFarland standard and diluting until a concentration of 1 × 10^3^ CFU/mL is reached. After plating their serial dilutions and counting the CFU per plate, the inoculum was checked to calculate the number of CFU/mL.

#### 3.5.1. Antimicrobial Susceptibility Assays

The time–kill curves were evaluated in the presence of CaP or CaP-Vanillin (10 mg, 50 mg, and 100 mg) in a volume of 2 mL of PBS. Bacteria were incubated at room temperature with agitation (120 rpm), and the number of CFU/mL at selected time intervals (0, 1, 2, 3, 4, 6, 8, 24 and 48 h) was determined. For this purpose, for each time-point, ten-fold serial dilutions were performed and 100 μL of these were spread in TSA plates and after 24 h incubation at 37 °C the number of colonies was counted, and CFU/mL calculated. The bactericidal effect was defined as ≥ 3-log10 CFU/mL (corresponding to 99.9% killing) decrease in comparison with the level for the initial inoculum [61]. For each time and dilution, three experimental and technical replicates were performed.

#### 3.5.2. Biofilm Formation Assays

The ability of CaP-Vanillin to prevent biofilm formation was quantified. Biofilm was formed on CaP and CaP-Vanillin in a sterile flat-bottomed 24-well polystyrene microtiter plate (Sarstedt, Nümbrecht, Germany). Wells were filled with 1 mL of TSB supplemented with 0.25% d-(+)-glucose (TSBG) and CaP or CaP-Vanillin at concentrations of 50 mg/mL or 100 mg/mL and 20 μL of the stock inoculum suspension, providing 1 × 10^4^ CFU/mL. After 48 h of incubation at 37 °C, both CaP and CaP–Vanillin samples were aseptically transferred into tubes and rinsed twice with sterile PBS to eliminate non-adherent cells. Subsequently, 1 mL of sterile TSB was added to each tube. The samples were then processed by vortexing for 1 min at 2500 rpm, followed by sonication for 1 min at 50 kHz, and an additional vortexing step for 1 min at 2500 rpm. To determine the number of viable bacteria attached to the granules, 100 µL of the resulting suspension and 100 µL of its ten-fold serial dilutions were plated on TSA and incubated at 37 °C for 24 h. Colonies were counted, and CFU/mL values were calculated. Three experimental and technical replicates were performed.

#### 3.5.3. Scanning Confocal Laser Microscopy

Scanning confocal laser microscopy determined cells’ viability in the biofilm in the presence of CaP or CaP-Vanillin. For it, 24-well plates (Sarstedt), equipped with sterile glass disks, were filled with 1 mL of TSBG, inoculated with 20 μL of the inoculum suspension with final inoculum of approximately 1 × 10^3^ CFU/mL and was grown for 24 h at 37 °C in presence of CaP or CaP-Vanillin. After three washes with 500 µL of sterile PBS, bacterial cells were stained using the Invitrogen™ LIVE/DEAD™ BacLight™ Bacterial Viability kit (Fisher Scientific, Madrid, Spain) according to the manufacturer’s protocol. This staining system combines SYTO^®^ 9 and propidium iodide, enabling discrimination between viable cells with intact membranes (green fluorescence) and cells with compromised membranes, considered dead or dying (red fluorescence). Confocal microscopy was performed on a Leica SP5 system (Leica, Wetzlar, Germany) in sequential mode using a 40× oil immersion objective. Excitation wavelengths were set at 480 nm for SYTO^®^ 9 and 490 nm for propidium iodide, with emission collected at 500 nm and 635 nm, respectively. Representative images were selected from at least three different regions per slide. All experiments included three biological and technical replicates to ensure reproducibility, and results are reported as mean ± SD.

### 3.6. In Vivo Study

The experimental protocols were approved by the Research Ethical Committee of Universitat Politécnica de València (ref. P05_07_05_20). Twelve female New Zealand rabbits (Granja Riera, L’Atmetlla del Vallés, Spain) with a body weight of 3.76 ± 0.25 kg were housed under controlled conditions and supplied with a standard diet and water ad libitum. After a quarantine period, animals were submitted to surgery to place the experimental (CaP-Vanillin) and control (CaP) materials in the median region of the distal femoral condyle. General anaesthesia was induced with an intramuscular injection of 3.12 mg/kg xylazine and 17.5 mg/kg ketamine. During surgery, anaesthesia was maintained with continuous intravenous administration of 21 mg/kg/h of Propofol. In sterile conditions, longitudinal incisions on the medial region of the distal femoral condyle were performed bilaterally. Bone defects of 4 mm in diameter and 6 mm in depth were realised with a drill, flushing and cooling with sterile 0.9% NaCl to remove bone debris. Next, 0.055 g of Cap and 0.055 g of CaP-Vanillin were weighed using a precision balance (PB-S/FACT/1129392386, Mettler Toledo) in a sterile Eppendorf and implanted into the right or left lateral femoral condyles. The surgical wound was closed routinely. All animals received 1 mg/kg of flunixin meglumine and 0.03 mg/kg of buprenorphine (3 days), and the surgical wounds were observed for healing and possible complications. No antimicrobial treatment was administered. Animals were divided into four groups depending on their group and experimentation time (Table 2).

Under general anaesthesia, at 2 and 8 weeks, animals were pharmacologically euthanized with intravenous injection of 5 mL sodium pentobarbital.

### 3.7. Histological Evaluation and Explanted Distal Femurs Histomorphometric Measurements

Bone segments were dehydrated in a graded series of alcohol/water mixture and embedded in TECHNOVIT resin (TECHNOVIT 7200 VLC Solution–Komponent 1:1000 m). Specimens were sectioned using EXAKT system, obtaining two sections of each specimen. Sections were stained with Mason-Goldner stain for histological evaluations and histomorphometric measurements performed by FIJI software (ImageJ 1.53c) [62]. For each section, regions of interest (ROI) were created: ROI corresponding to a region defined by material’s perimeter This ROI were defined to measure bone formation between the implanted material. The following histomorphometric parameter was defined:

Bone area inside (%): measured as the amount of bone found in ROI, and calculated by the following formula:

(1) $$Bone\;area\;inside\left(\%\right)=\frac{Bone_{ROI1}}{Total\;Area_{ROI}-Area_{implant}}\cdot 100$$

**Figure 7:**
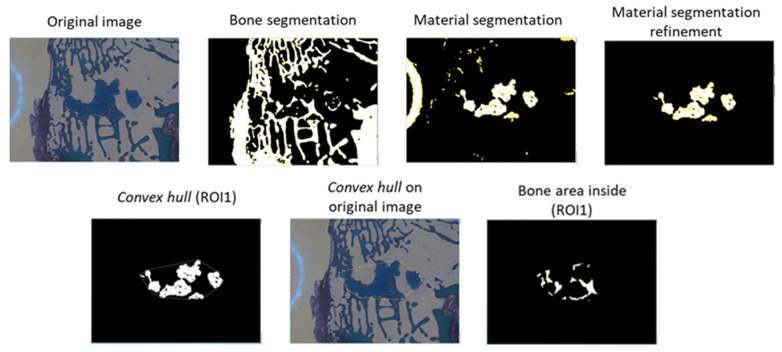
Images of the various steps involved in processing histological sections. All images were taken at 100× magnification.

Figure 7 shows the procedure followed to obtain bone area inside.

### 3.8. Statistical Analysis

Statistical analysis was performed using R software (version 4.1.3). A two-factor analysis (Functionalization × Time) was implemented to evaluate bone regeneration. Homogeneity of variance was assessed using Levene’s test. For comparisons between groups with equal variances, Student’s *t*-test was used; for groups with unequal variances, Welch’s *t*-test was applied. Results are reported with exact *p*-values, 95% Confidence Intervals (CI), and mean differences as a measure of effect size.

## 4. Conclusions

Vanillin-functionalized CaP scaffolds (CaP-Vanillin) were developed via the covalent anchoring of the vanillin derivative **3** to commercially available CaP scaffolds. TGA studies proved the correct functionalization of the material. Besides, HR-FESEM demonstrated that no substantial changes in the morphology of the materials at the micrometric level were observed after the functionalization process. CaP-Vanillin shows to be a remarkable in vitro antibacterial against the bacterium *S. epidermidis*. Experiments showed that no viable cells were detected at CaP-Vanillin concentrations of 100 mg/mL, 50 mg/mL, and 10 mg/mL after 7 h, 8 h and 48 h, respectively. Furthermore, CaP-Vanillin showed a high ability to reduce biofilm formation after 48 h of incubation with *S. epidermidis* RP62A. CaP-Vanillin achieved a 7-log reduction in viable biofilm cells of *S. epidermidis* compared to CaP at a concentration of 100 mg/mL, producing a 2-log reduction at a concentration of 50 mg/mL. The mean lethality of *S. epidermidis* in the biofilm produced by CaP-Vanillin was 98.9% and >99.9% at a concentration of 50 mg/mL and 100 mg/mL, respectively, compared to CaP. In addition, in vivo studies with CaP-Vanillin and CaP placed in the median region of the distal femoral condyle on New Zealand female rabbits were also carried out, to assess if CaP-Vanillin interferes with osseointegration. Regarding the time course, CaP-Vanillin shows a significant growth of bone area between 2 and 8 weeks, resulting in a comparable amount of bone growth to under control conditions, which demonstrates that vanillin does not hinder bone growth. In summary, results showed that the material exhibits an excellent safety profile and does not impede bone growth, maintaining its antimicrobial potential demonstrated in vitro. Considering this and the excellent in vitro antimicrobial properties of CaP-Vanillin, we are conducting further studies to state the behaviour of the CaP-Vanillin implant in terms of infection inhibition and the potential application of vanillin and other EOC coatings in medical devices for biomedical applications.

## Figures and Tables

**Table 1:** Histomorphometric results for bone area inside percentage for each group (*n* = 6).

Comparison Factor	Mean ± SD (%)	95% Confidence Interval (CI)	Mean Difference (Effect Size)	*p*-Value
2 weeks: CaP vs. CaP-Vanillin	30.65 ± 11.13 vs. 23.48 ± 9.25	[−15.82, 1.50]	−7.16	0.101
8 weeks: CaP vs. CaP-Vanillin	33.05 ± 7.11 vs. 31.56 ± 7.24	[−7.56, 4.59]	−1.48	0.618
Temporal CaP: 2w vs. 8w	30.65 ± 11.13 vs. 33.05 ± 7.11	[−11.45, 6.64]	2.40	0.535
Temporal CaP-Vanillin: 2w vs. 8w	23.48 ± 9.25 vs. 31.56 ± 7.24	[0.98, 15.18]	8.08	0.0263

**Table 2:** Experimental design.

	2 Weeks	8 Weeks
CaP group	6 samples	6 samples
CaP-Vanillin group	6 samples	6 samples
TOTAL	12 samples (6 animals)	12 samples (6 animals)

## Data Availability

The original contributions presented in this study are included in the article/Supplementary Materials. Further inquiries can be directed to the corresponding authors.

## Supplementary Materials

The following supporting information can be downloaded at: https://www.mdpi.com/article/10.3390/ph19010091/s1. NMR analysis.

## References

[1] Hench L.L., Thompson I. (2010). Twenty-first century challenges for biomaterials. J. R. Soc. Interface.

[2] Daculsi G. (1998). Biphasic calcium phosphate concept applied to artificial bone, implant coating and injectable bone substitute. Biomaterials.

[3] Baino F., Novajra G., Vitale-Brovarone C. (2015). Bioceramics and scaffolds: A winning combination for tissue engineering. Front. Bioeng. Biotechnol..

[4] Ferguson J., Diefenbeck M., Mcnally M. (2017). Ceramic Biocomposites as Biodegradable Antibiotic Carriers in the Treatment of Bone Infections. J. Bone Jt. Infect..

[5] Eliaz N., Metoki N. (2017). Calcium phosphate bioceramics: A review of their history, structure, properties, coating technologies and biomedical applications. Materials.

[6] Kattimani V.S., Kondaka S., Lingamaneni K.P. (2016). Hydroxyapatite–-Past, Present, and Future in Bone Regeneration. Bone Tissue Regen. Insights.

[7] Uskoković V., Wu V.M. (2016). Calcium phosphate as a key material for socially responsible tissue engineering. Materials.

[8] Huzum B., Puha B., Necoara R., Gheorghevici S., Puha G., Filip A., Sirbu P., Alexa O. (2021). Biocompatibility assessment of biomaterials used in orthopedic devices: An overview (Review). Exp. Ther. Med..

[9] Lane J.M., Mait J.E., Unnanuntana A., Hirsch B.P., Shaffer A.D., Shonuga O.A. (2011). Materials in Fracture Fixation. Comprehensive Biomaterials.

[10] Choi A.H., Ben-Nissan B., Conway R.C., Macha I.J. (2014). Advances in Calcium Phosphate Nanocoatings and Nanocomposites.

[11] de Groot K., Wolke J.G.C., Jansen J.A. (1998). Calcium phosphate coatings for medical implants. Proc. Inst. Mech. Eng. Part H J. Eng. Med..

[12] Esteban J., Cordero-Ampuero J. (2011). Treatment of prosthetic osteoarticular infections. Expert Opin. Pharmacother..

[13] Healy W.L., Della Valle C.J., Iorio R., Berend K.R., Cushner F.D., Dalury D.F., Lonner J.H. (2013). Complications of total knee arthroplasty: Standardized list and definitions of the knee society knee. Clin. Orthop. Relat. Res..

[14] Arciola C.R., Visai L., Testoni F., Arciola S., Campoccia D., Speziale P., Montanaro L. (2011). Concise survey of *Staphylococcus aureus* virulence factors that promote adhesion and damage to peri-implant tissues. Int. J. Artif. Organs.

[15] Tande A.J., Patel R. (2014). Prosthetic joint infection. Clin. Microbiol. Rev..

[16] Sharma S., Mohler J., Mahajan S.D., Schwartz S.A., Bruggemann L., Aalinkeel R. (2023). Microbial Biofilm: A Review on Formation, Infection, Antibiotic Resistance, Control Measures, and Innovative Treatment. Microorganisms.

[17] Widerström M., Stegger M., Johansson A., Gurram B.K., Larsen A.R., Wallinder L., Edebro H., Monsen T. (2022). Heterogeneity of *Staphylococcus epidermidis* in prosthetic joint infections: Time to reevaluate microbiological criteria?. Eur. J. Clin. Microbiol. Infect. Dis..

[18] Tomizawa T., Ishikawa M., Bello-Irizarry S.N., de Mesy Bentley K.L., Ito H., Kates S.L., Daiss J.L., Beck C., Matsuda S., Schwarz E.M. (2020). Biofilm Producing *Staphylococcus epidermidis* (RP62A Strain) Inhibits Osseous Integration Without Osteolysis and Histopathology in a Murine Septic Implant Model. J. Orthop. Res..

[19] Guarch-Pérez C., Riool M., de Boer L., Kloen P., Zaat S.A.J. (2023). Bacterial reservoir in deeper skin is a potential source for surgical site and biomaterial-associated infections. J. Hosp. Infect..

[20] Severn M.M., Horswill A.R. (2023). *Staphylococcus epidermidis* and its dual lifestyle in skin health and infection. Nat. Rev. Microbiol..

[21] Inzana J.A., Schwarz E.M., Kates S.L., Awad H.A. (2016). Biomaterials approaches to treating implant-associated osteomyelitis. Biomaterials.

[22] Mas N., Galiana I., Hurtado S., Mondragón L., Bernardos A., Sancenón F., Marcos M.D., Amorós P., Abril-Utrillas N., Martínez-Máñez R. (2014). Enhanced antifungal efficacy of tebuconazole using gated pH-driven mesoporous nanoparticles. Int. J. Nanomed..

[23] Polo L., Gómez-Cerezo N., Aznar E., Vivancos J.L., Sancenón F., Arcos D., Vallet-Regí M., Martínez-Máñez R. (2017). Molecular gates in mesoporous bioactive glasses for the treatment of bone tumors and infection. Acta Biomater..

[24] Davies D. (2003). Understanding biofilm resistance to antibacterial agents. Nat. Rev. Drug Discov..

[25] Sadekuzzaman M., Yang S., Mizan M.F.R., Ha S.D. (2015). Current and Recent Advanced Strategies for Combating Biofilms. Compr. Rev. Food Sci. Food Saf..

[26] Freires I., Denny C., Benso B., de Alencar S., Rosalen P. (2015). Antibacterial Activity of Essential Oils and Their Isolated Constituents against Cariogenic Bacteria: A Systematic Review. Molecules.

[27] Chávez-González M.L., Rodríguez-Herrera R., Aguilar C.N. (2016). Essential Oils: A Natutal Alternative to Combat Antibiotics Resistance. Antibiotic Resistance, Mechanisms and New Antimicrobial Approaches.

[28] Savoia D. (2012). Plant-derived antimicrobial compounds: Alternatives to antibiotics. Future Microbiol..

[29] Bassolé I.H.N., Juliani H.R. (2012). Essential Oils in Combination and Their Antimicrobial Properties. Molecules.

[30] Osaili T.M., Dhanasekaran D.K., Zeb F., Faris M.E., Naja F., Radwan H., Cheikh Ismail L., Hasan H., Hashim M., Obaid R.S. (2023). A Status Review on Health-Promoting Properties and Global Regulation of Essential Oils. Molecules.

[31] Bernardos A., Bozik M., Alvarez S., Saskova M., Perez-Esteve E., Kloucek P., Lhotka M., Frankova A., Martinez-Manez R. (2019). The efficacy of essential oil components loaded into montmorillonite against Aspergillus niger and *Staphylococcus aureus*. Flavour Fragr. J..

[32] Bagherifard S. (2017). Mediating bone regeneration by means of drug eluting implants: From passive to smart strategies. Mater. Sci. Eng. C.

[33] Giacomini D., Torricelli P., Gentilomi G.A., Boanini E., Gazzano M., Bonvicini F., Benetti E., Soldati R., Martelli G., Rubini K. (2017). Monocyclic β-lactams loaded on hydroxyapatite: New biomaterials with enhanced antibacterial activity against resistant strains. Sci. Rep..

[34] Mas N., Arcos D., Polo L., Aznar E., Sánchez-Salcedo S., Sancenón F., García A., Marcos M.D., Baeza A., Vallet-Regí M. (2014). Towards the Development of Smart 3D “Gated Scaffolds” for On-Command Delivery. Small.

[35] Ribes S., Ruiz-Rico M., Pérez-Esteve É., Fuentes A., Talens P., Martínez-Máñez R., Barat J.M. (2017). Eugenol and thymol immobilised on mesoporous silica-based material as an innovative antifungal system: Application in strawberry jam. Food Control.

[36] Ruiz-Rico M., Pérez-Esteve É., Bernardos A., Sancenón F., Martínez-Máñez R., Marcos M.D., Barat J.M. (2017). Enhanced antimicrobial activity of essential oil components immobilized on silica particles. Food Chem..

[37] Gomes F., Teixeira P., Oliveira R. (2014). Mini-review: *Staphylococcus epidermidis* as the most frequent cause of nosocomial infections: Old and new fighting strategies. Biofouling.

[38] Ziebuhr W., Hennig S., Eckart M., Kränzler H., Batzilla C., Kozitskaya S. (2006). Nosocomial infections by *Staphylococcus epidermidis*: How a commensal bacterium turns into a pathogen. Int. J. Antimicrob. Agents.

[39] Guarino V., Causa F., Netti P.A., Ciapetti G., Pagani S., Martini D., Baldini N., Ambrosio L. (2008). The role of hydroxyapatite as solid signal on performance of PCL porous scaffolds for bone tissue regeneration. J. Biomed. Mater. Res. Part B Appl. Biomater..

[40] Degirmenbasi N., Kalyon D.M., Birinci E. (2006). Biocomposites of nanohydroxyapatite with collagen and poly(vinyl alcohol). Colloids Surf. B Biointerfaces.

[41] Gogia J.S., Meehan J.P., Di Cesare P.E., Jamali A.A. (2009). Local antibiotic therapy in osteomyelitis. Semin. Plast. Surg..

[42] McConoughey S.J., Howlin R., Granger J.F., Manring M.M., Calhoun J.H., Shirtliff M., Kathju S., Stoodley P. (2014). Biofilms in periprosthetic orthopedic infections. Future Microbiol..

[43] Arya S.S., Rookes J.E., Cahill D.M., Lenka S.K. (2021). Vanillin: A review on the therapeutic prospects of a popular flavouring molecule. Adv. Tradit. Med..

[44] Fuentes C., Fuentes A., Barat J.M., Ruiz M.J. (2021). Relevant essential oil components: A minireview on increasing applications and potential toxicity. Toxicol. Mech. Methods.

[45] Héquet A., Humblot V., Berjeaud J.-M., Pradier C.-M. (2011). Optimized grafting of antimicrobial peptides on stainless steel surface and biofilm resistance tests. Colloids Surf. B Biointerfaces.

[46] Sadowska J.M., Genoud K.J., Kelly D.J., O’Brien F.J. (2021). Bone biomaterials for overcoming antimicrobial resistance: Advances in non-antibiotic antimicrobial approaches for regeneration of infected osseous tissue. Mater. Today.

[47] Boanini E., Cassani M., Rubini K., Boga C., Bigi A. (2018). (9R)-9-Hydroxystearate-Functionalized Anticancer Ceramics Promote Loading of Silver Nanoparticles. Nanomaterials.

[48] Silingardi F., Bonvicini F., Cassani M.C., Mazzaro R., Rubini K., Gentilomi G.A., Bigi A., Boanini E. (2022). Hydroxyapatite Decorated with Tungsten Oxide Nanoparticles: New Composite Materials against Bacterial Growth. J. Funct. Biomater..

[49] Dahiya A., Chaudhari V.S., Kushram P., Bose S. (2024). 3D Printed SiO2 –Tricalcium Phosphate Scaffolds Loaded with Carvacrol Nanoparticles for Bone Tissue Engineering Application. J. Med. Chem..

[50] Alves A.P.N., Arango-Ospina M., Oliveira R.L.M.S., Ferreira I.M., de Moraes E.G., Hartmann M., de Oliveira A.P.N., Boccaccini A.R., de Sousa Trichês E. (2023). 3D-printed β-TCP/S53P4 bioactive glass scaffolds coated with tea tree oil: Coating optimization, in vitro bioactivity and antibacterial properties. J. Biomed. Mater. Res. Part B Appl. Biomater..

[51] Lallukka M., Gamna F., Gobbo V.A., Prato M., Najmi Z., Cochis A., Rimondini L., Ferraris S., Spriano S. (2022). Surface Functionalization of Ti6Al4V-ELI Alloy with Antimicrobial Peptide Nisin. Nanomaterials.

[52] Ghasemi F., Jahani A., Moradi A., Ebrahimzadeh M.H., Jirofti N. (2023). Different Modification Methods of Poly Methyl Methacrylate (PMMA) Bone Cement for Orthopedic Surgery Applications. Arch. Bone Jt. Surg..

[53] Zegre M., Poljańska E., Caetano L.A., Gonçalves L., Bettencourt A. (2023). Research progress on biodegradable polymeric platforms for targeting antibiotics to the bone. Int. J. Pharm..

[54] Perez R.A., Kim H.-W., Ginebra M.-P. (2012). Polymeric additives to enhance the functional properties of calcium phosphate cements. J. Tissue Eng..

[55] Beeching N.J., Thomas M.G., Roberts S., Lang S.D.R. (1986). Comparative in-vitro activity of antibiotics incorporated in acrylic bone cement. J. Antimicrob. Chemother..

[56] Jiang J.-L., Li Y.-F., Fang T.-L., Zhou J., Li X.-L., Wang Y.-C., Dong J. (2012). Vancomycin-loaded nano-hydroxyapatite pellets to treat MRSA-induced chronic osteomyelitis with bone defect in rabbits. Inflamm. Res..

[57] Shi P., Zuo Y., Li X., Zou Q., Liu H., Zhang L., Li Y., Morsi Y.S. (2010). Gentamicin-impregnated chitosan/nanohydroxyapatite/ethyl cellulose microspheres granules for chronic osteomyelitis therapy. J. Biomed. Mater. Res. Part A.

[58] Amna T., Alghamdi A.A.A., Shang K., Hassan M.S. (2021). Nigella Sativa-Coated Hydroxyapatite Scaffolds: Synergetic Cues to Stimulate Myoblasts Differentiation and Offset Infections. Tissue Eng. Regen. Med..

[59] Polo L., Díaz de Greñu B., Della Bella E., Pagani S., Torricelli P., Vivancos J.L., Ruiz-Rico M., Barat J.M., Aznar E., Martínez-Máñez R. (2018). Antimicrobial activity of commercial calcium phosphate based materials functionalized with vanillin. Acta Biomater..

[60] Yun H.-M., Kim E., Kwon Y.-J., Park K.-R. (2024). Vanillin Promotes Osteoblast Differentiation, Mineral Apposition, and Antioxidant Effects in Pre-Osteoblasts. Pharmaceutics.

[61] Zadrazilova I., Pospisilova S., Pauk K., Imramovsky A., Vinsova J., Cizek A., Jampilek J. (2015). In Vitro Bactericidal Activity of 4- and 5-Chloro-2-hydroxy-N-[1-oxo-1-(phenylamino)alkan-2-yl]benzamides against MRSA. BioMed Res. Int..

[62] Schindelin J., Arganda-Carreras I., Frise E., Kaynig V., Longair M., Pietzsch T., Preibisch S., Rueden C., Saalfeld S., Schmid B. (2012). Fiji: An open-source platform for biological-image analysis. Nat. Methods.

