# Supplementary material for: In Vivo Study of Osseointegrable Bone Calcium Phosphate (CaP) Implants Coated with a Vanillin Derivative
Source: Pharmaceuticals (Basel). 2026 Jan 3;19(1):91. doi: 10.3390/ph19010091 (PMC12844627; doi:10.3390/ph19010091)
Supplement: Supplementary file 1 [file pharmaceuticals-19-00091-s001.zip › pharmaceuticals-4041153-supplementary.pdf]

Supplementary Material

*NMR data of vanillin derivative (**3**)*

^{1}H NMR (MeOD): δ = 6.8 (1H, s); 6.62 (2H, s); 3.72 (3H, s); 3.52 (2H, m); 2.63–2.35 (4H, m); 1.46–1,30 (4H, m), 1.28–1.18 (2H, m) ppm.

^{13}C NMR (MeOD): δ = 149.11; 147.14, 131.59; 122.41; 116.10; 113.29; 65.33; 56.33, 54.27; 42.16; 32.89; 30.05; 25.66 ppm.
